# Hexaaqua­iron(II) bis­[*fac*-tribromido­tricarbonyl­ferrate(II)]

**DOI:** 10.1107/S1600536809036198

**Published:** 2009-09-12

**Authors:** Eva Becker, Karl Kirchner, Kurt Mereiter

**Affiliations:** a Institute of Applied Synthetic Chemistry, Vienna University of Technology, Getreidemarkt 9/163, A-1060 Vienna, Austria; b Institute of Chemical Technologies and Analytics, Vienna University of Technology, Getreidemarkt 9/164SC, A-1060 Vienna, Austria

## Abstract

In the title compound, [Fe(H_2_O)_6_][FeBr_3_(CO)_3_]_2_, both Fe atoms have an octa­hedral coordination and the bromide carbonyl complex has a *fac*-stereochemistry. The [Fe(H_2_O)_6_]^2+^ octa­hedron has point symmetry 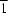 and is slightly compressed along one O—Fe—O axis. The [FeBr_3_(CO)_3_]^−^ anion has point symmetry 1 and mean bond lengths of Fe—Br = 2.455 (5) Å and Fe—C = 1.809 (2) Å. The cation and anion complexes are mutually linked *via* O—H⋯Br hydrogen bonds with O⋯Br distances of 3.340 (3) to 3.388 (3) Å.

## Related literature

For the chemistry of FeBr_2_(CO)_4_, see: Hieber & Bader (1928 ▶); Robertson *et al.* (2000 ▶). For the syntheses and crystal structures of anologous compounds with [(Fe/Co/Ru)(H_2_O)_6_]^2+^ cations and [(Ru/Os)(Cl/I)_3_(CO)_3_]^−^ complexes, see: Allen (2002 ▶); Taimisto *et al.* (2003 ▶); Haukka *et al.* (2006 ▶); Jakonen *et al.* (2007 ▶).
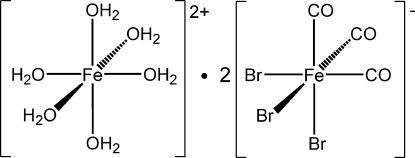


## Experimental

### 

#### Crystal data

[Fe(H_2_O)_6_][FeBr_3_(CO)_3_]_2_
*M* *_r_* = 923.17
Orthorhombic, 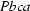

*a* = 11.9334 (8) Å
*b* = 9.3394 (6) Å
*c* = 20.5775 (14) Å
*V* = 2293.4 (3) Å^3^
*Z* = 4
Mo *K*α radiation
μ = 12.37 mm^−1^
*T* = 100 K
0.26 × 0.10 × 0.08 mm

#### Data collection

Bruker SMART APEX CCD diffractometer
Absorption correction: multi-scan (*SADABS*; Bruker, 2003 ▶) *T*_min_ = 0.15, *T*_max_ = 0.37
24075 measured reflections
3316 independent reflections
2674 reflections with *I* > 2σ(*I*)
*R*_int_ = 0.061

#### Refinement

*R*[*F* ^2^ > 2σ(*F* ^2^)] = 0.034
*wR*(*F* ^2^) = 0.070
*S* = 1.04
3316 reflections
145 parameters
18 restraints
H atoms treated by a mixture of independent and constrained refinement
Δρ_max_ = 0.98 e Å^−3^
Δρ_min_ = −0.68 e Å^−3^

### 

Data collection: *SMART* (Bruker, 2003 ▶); cell refinement: *SAINT* (Bruker, 2003 ▶); data reduction: *SAINT* and *XPREP* (Bruker, 2003 ▶); program(s) used to solve structure: *SHELXS97* (Sheldrick, 2008 ▶); program(s) used to refine structure: *SHELXL97* (Sheldrick, 2008 ▶); molecular graphics: *SHELXTL* (Sheldrick, 2008 ▶); software used to prepare material for publication: *SHELXTL*.

## Supplementary Material

Crystal structure: contains datablocks global, I. DOI: 10.1107/S1600536809036198/om2275sup1.cif

Structure factors: contains datablocks I. DOI: 10.1107/S1600536809036198/om2275Isup2.hkl

Additional supplementary materials:  crystallographic information; 3D view; checkCIF report

## Figures and Tables

**Table 1:** Selected bond lengths (Å)

Fe1—O1*W*	2.137 (3)
Fe1—O2*W*	2.128 (3)
Fe1—O3*W*	2.098 (3)
Fe2—C1	1.812 (4)
Fe2—C2	1.807 (4)
Fe2—C3	1.808 (4)
Fe2—Br1	2.4479 (6)
Fe2—Br2	2.4605 (6)
Fe2—Br3	2.4558 (6)

**Table 2:** Hydrogen-bond geometry (Å, °)

*D*—H⋯*A*	*D*—H	H⋯*A*	*D*⋯*A*	*D*—H⋯*A*
O1*W*—H1*A*⋯Br1	0.821 (19)	2.52 (2)	3.340 (3)	174 (4)
O1*W*—H1*B*⋯Br2^i^	0.821 (19)	2.69 (2)	3.475 (3)	162 (5)
O2*W*—H2*A*⋯Br2^ii^	0.821 (19)	2.55 (2)	3.373 (3)	177 (5)
O2*W*—H2*B*⋯Br2^i^	0.821 (19)	2.56 (2)	3.341 (3)	160 (5)
O3*W*—H3*A*⋯Br3	0.821 (19)	2.58 (2)	3.388 (3)	169 (5)
O3*W*—H3*B*⋯Br3^iii^	0.821 (19)	2.53 (2)	3.346 (3)	177 (4)

Symmetry codes: (i) 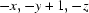; (ii) 
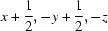
; (iii) 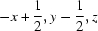.

**Fig. 1.**
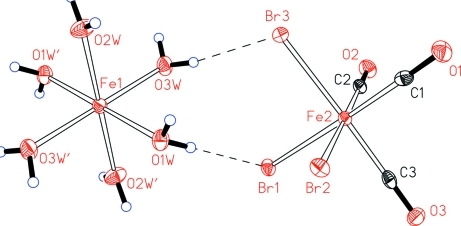
Perspective view of the title compound with the atom numbering scheme. Atoms with primed labels are generated by inversion (-x,-y,-z). Displacement ellipsoids are at the 50% probability level.

**Fig. 2.**
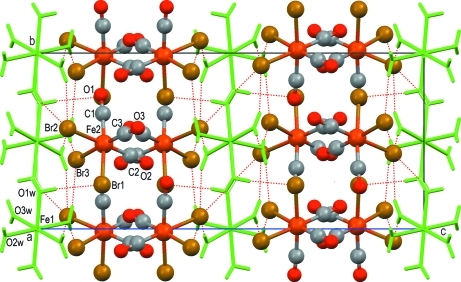
Packing diagram of the title compound viewed down the a axis. [Fe(H2O)6] octahedra in green and stick bond representation, [FeBr3(CO)3] in ball-and-stick representation, hydrogen bonds shown as red broken lines.

## supplementary crystallographic information

### Comment

FeBr_2_(CO)_4_, easily accessible by reaction of Fe(CO)_5_ with bromine (Hieber & Bader, 1928; Robertson *et al.*, 2000), is stable under dry conditions but reacts readily with various protic or coordinating solvents and thus turned out to be an interesting precursor for iron complexes which are otherwise difficult to obtain. By treatment of FeBr_2_(CO)_4_ with wet benzene the title compound was obtained according to the equation: 3 FeBr_2_(CO)_4_ + 6H_2_O → Fe(H_2_O)_6_ + 2 FeBr_3_(CO)_3_ + 6 CO. The structure (Fig. 1) contains a [Fe(H_2_O)_6_]^2+^ octahedron with point symmetry 1 and a [FeBr_3_(CO)_3_]^-^ octahedron with iron with point symmetry 1 (Fig. 1). The Fe—O bond lengths in [Fe(H_2_O)_6_]^2+^ (Table 1) correspond well with high-spin iron(II) in an axially weakly compressed octahedron. The [FeBr_3_(CO)_3_]^-^ anion has a facial disposition of the bromide and carbonyl ligands and shows each three very uniform Fe—Br and Fe—C bond distances with mean values of Fe—Br = 2.455 (5) Å and Fe—C = 1.809 (2) Å (Table 1) consistent with a low-spin state. The bond angles about this iron deviate up to 5.6° from 90 and 180°. C—O bond lengths average to 1.128 (1) Å, and Fe—C—O angles 177.9 (10)°. Each of the three independent water molecules is coordinated by Fe1 and by pair of bromine atoms as hydrogen bond acceptors. Their coordination environments are pyramidal to flat pyramidal (O2w). The six independent O—H···Br bonds (Table 2) are quite uniform in distances and angles, all being essentially linear. The structure has various architectural aspects, but is best regarded as consisting of double layers of [FeBr_3_(CO)_3_] octahedra held together by interactions between the mainly inward-oriented CO groups whereas the bromine atoms define their outer surfaces (Fig. 2). These double layers are oriented parallel to (100) and centered at *y* = 1/4 and 3/4. Intercalated between these double layers are single layers of [Fe(H_2_O)_6_]^2+^ octahedra at *z* = 0, 1/2 and 1, which bridge the double layers *via* the O—H···Br hydrogen bonds. The title compound has no precedents in iron structural chemistry, but a few analogous [*MX*_3_(CO)_3_]^-^ complexes are known for *M* = Ru, Os, Re, and *X* = Cl, Br, I (Cambridge Structural Database - CSD; Version 5.30 of November 2008; Allen, 2002). Most related to the title compound is [Ru(H_2_O)_6_]^2+^[RuCl_3_(CO)_3_]^-^_2_.2H_2_O (Taimisto *et al.*, 2003), obtained from [RuCl_2_(CO)_3_]_2_ in wet CH_2_Cl_2_. It has, despite being triclinic (title compound is orthorhombic), an additional uncoordinated H_2_O, a structural architecture analogous to the title compound. More recently, this formerly unique compound was expanded into three isomorphous series of type 1, [(Fe/Co)(H_2_O)_6_]^2+^[(Ru/Os)Cl_3_(CO)_3_]^-^_2_, triclinic, space group *P*1, of type 2, [(Ru/Fe/Co)(H_2_O)_6_]^2+^[(Ru/Os)Cl_3_(CO)_3_]^-^_2_.2H_2_O, triclinic, space group *P*1, and of type 3, [(Fe/Co)(H_2_O)_6_]^2+^[RuI_3_(CO)_3_]^-^_2_.2H_2_O, monoclinic, space group *P*2_1_/*c* (Haukka *et al.*, 2006; Jakonen *et al.*, 2007). All these compounds share the [*MX*_3_(CO)_3_]^-^ (*X* = Cl, Br, I) double layer plus [*M*(H_2_O)_6_]^2+^ single layer architecture of the title compound with additional non-coordinated water molecules (structure types 2 and 3) being involved in the [*M*(H_2_O)_6_]^2+^ layers.

### Experimental

The title compound was synthesized as follows: FeBr_2_(CO)_4_ (200 mg, 0.610 mmol; Hieber & Bader, 1928) was dissolved in wet benzene (20 ml, containing 1.67 mmol water) resulting in significant CO gas evolution. The solution was allowed to stand for 15 minutes until no further gas evolution was observed. The solution was set aside for about 1 h to give orange crystals of the title copound. Yield: 75 mg (40%).

### Refinement

All H atoms belong to H_2_O molecules. They were refined in *x*,*y*,*z* using hard SADI restraints of program *SHELXL97* (Sheldrick, 2008) to make all O—H bond lengths and all intramolecular H—H distances equal. Moreover, pairs of identical *U*_iso_(H) values were refined for each water molecule.

### Crystal data

**Table:**

[Fe(H_2_O)_6_][FeBr_3_(CO)_3_]_2_	*F*(000) = 1728
*M**_r_* = 923.17	*D*_x_ = 2.674 Mg m^−^^3^
Orthorhombic, *P**b**c**a*	Mo *K*α radiation, λ = 0.71073 Å
Hall symbol: -P 2ac 2ab	Cell parameters from 4102 reflections
*a* = 11.9334 (8) Å	θ = 2.6–29.5°
*b* = 9.3394 (6) Å	µ = 12.37 mm^−^^1^
*c* = 20.5775 (14) Å	*T* = 100 K
*V* = 2293.4 (3) Å^3^	Prism, brown
*Z* = 4	0.26 × 0.10 × 0.08 mm

### Data collection

**Table:**

Bruker SMART APEX CCD diffractometer	3316 independent reflections
Radiation source: fine-focus sealed tube	2674 reflections with *I* > 2σ(*I*)
graphite	*R*_int_ = 0.061
ω and φ scans	θ_max_ = 30.0°, θ_min_ = 2.6°
Absorption correction: multi-scan (*SADABS*; Bruker, 2003)	*h* = −16→16
*T*_min_ = 0.15, *T*_max_ = 0.37	*k* = −12→12
24075 measured reflections	*l* = −28→28

### Refinement

**Table:**

Refinement on *F*^2^	Primary atom site location: structure-invariant direct methods
Least-squares matrix: full	Secondary atom site location: difference Fourier map
*R*[*F*^2^ > 2σ(*F*^2^)] = 0.034	Hydrogen site location: inferred from neighbouring sites
*wR*(*F*^2^) = 0.070	H atoms treated by a mixture of independent and constrained refinement
*S* = 1.04	*w* = 1/[σ^2^(*F*_o_^2^) + (0.0343*P*)^2^] where *P* = (*F*_o_^2^ + 2*F*_c_^2^)/3
3316 reflections	(Δ/σ)_max_ = 0.001
145 parameters	Δρ_max_ = 0.98 e Å^−^^3^
18 restraints	Δρ_min_ = −0.67 e Å^−^^3^

### Special details

**Table:**

Geometry. All e.s.d.'s (except the e.s.d. in the dihedral angle between two l.s. planes) are estimated using the full covariance matrix. The cell e.s.d.'s are taken into account individually in the estimation of e.s.d.'s in distances, angles and torsion angles; correlations between e.s.d.'s in cell parameters are only used when they are defined by crystal symmetry. An approximate (isotropic) treatment of cell e.s.d.'s is used for estimating e.s.d.'s involving l.s. planes.
Refinement. Refinement of *F*^2^ against ALL reflections. The weighted *R*-factor *wR* and goodness of fit *S* are based on *F*^2^, conventional *R*-factors *R* are based on *F*, with *F* set to zero for negative *F*^2^. The threshold expression of *F*^2^ > σ(*F*^2^) is used only for calculating *R*-factors(gt) *etc*. and is not relevant to the choice of reflections for refinement. *R*-factors based on *F*^2^ are statistically about twice as large as those based on *F*, and *R*- factors based on ALL data will be even larger.

### Fractional atomic coordinates and isotropic or equivalent isotropic displacement parameters (Å^2^)

**Table:**

	*x*	*y*	*z*	*U*_iso_*/*U*_eq_	
Fe1	0.0000	0.0000	0.0000	0.01291 (15)	
O1W	−0.0694 (2)	0.2102 (3)	0.00795 (12)	0.0182 (5)	
O2W	0.1316 (2)	0.0750 (3)	−0.06090 (15)	0.0232 (6)	
O3W	0.0994 (2)	0.0389 (3)	0.08220 (14)	0.0215 (6)	
H1A	−0.077 (4)	0.227 (5)	0.0468 (10)	0.054 (12)*	
H1B	−0.037 (4)	0.277 (4)	−0.010 (2)	0.054 (12)*	
H2A	0.194 (2)	0.039 (4)	−0.067 (2)	0.052 (12)*	
H2B	0.132 (4)	0.157 (3)	−0.075 (2)	0.052 (12)*	
H3A	0.104 (4)	0.124 (2)	0.091 (2)	0.043 (11)*	
H3B	0.161 (2)	0.001 (4)	0.086 (2)	0.043 (11)*	
Fe2	−0.00342 (4)	0.48304 (5)	0.17284 (2)	0.01109 (11)	
Br1	−0.09906 (3)	0.25138 (4)	0.168259 (17)	0.01518 (9)	
Br2	−0.11162 (3)	0.57039 (4)	0.079096 (17)	0.01582 (9)	
Br3	0.14630 (3)	0.39406 (4)	0.101623 (18)	0.01610 (9)	
C1	0.0671 (3)	0.6547 (4)	0.17014 (16)	0.0147 (7)	
O1	0.1100 (2)	0.7616 (3)	0.16509 (13)	0.0217 (6)	
C2	0.0777 (3)	0.4172 (4)	0.24072 (18)	0.0146 (7)	
O2	0.1272 (2)	0.3740 (3)	0.28314 (13)	0.0205 (6)	
C3	−0.1149 (3)	0.5406 (3)	0.22633 (18)	0.0147 (7)	
O3	−0.1840 (2)	0.5739 (3)	0.26062 (13)	0.0194 (5)	

### Atomic displacement parameters (Å^2^)

**Table:**

	*U*^11^	*U*^22^	*U*^33^	*U*^12^	*U*^13^	*U*^23^
Fe1	0.0130 (4)	0.0108 (3)	0.0149 (3)	0.0008 (3)	−0.0008 (3)	−0.0007 (3)
O1W	0.0231 (15)	0.0132 (12)	0.0182 (14)	0.0015 (11)	−0.0009 (11)	−0.0008 (10)
O2W	0.0206 (15)	0.0173 (13)	0.0317 (16)	0.0026 (12)	0.0083 (13)	0.0042 (12)
O3W	0.0220 (15)	0.0165 (13)	0.0260 (15)	0.0059 (11)	−0.0072 (12)	−0.0062 (11)
Fe2	0.0113 (2)	0.0102 (2)	0.0117 (2)	−0.00088 (19)	0.00038 (19)	−0.00026 (17)
Br1	0.01807 (18)	0.01203 (17)	0.01544 (17)	−0.00387 (13)	0.00183 (14)	−0.00048 (12)
Br2	0.01585 (18)	0.01523 (17)	0.01639 (17)	−0.00118 (13)	−0.00296 (14)	0.00300 (13)
Br3	0.01316 (18)	0.01727 (17)	0.01787 (18)	−0.00097 (14)	0.00263 (14)	−0.00377 (13)
C1	0.0114 (17)	0.0197 (18)	0.0131 (17)	0.0020 (14)	−0.0001 (13)	0.0008 (13)
O1	0.0247 (15)	0.0175 (14)	0.0229 (14)	−0.0057 (11)	0.0047 (11)	0.0014 (10)
C2	0.0141 (18)	0.0108 (15)	0.0188 (18)	−0.0040 (13)	0.0031 (14)	−0.0023 (13)
O2	0.0209 (14)	0.0191 (13)	0.0216 (14)	0.0000 (11)	−0.0051 (12)	0.0006 (10)
C3	0.0187 (19)	0.0087 (15)	0.0167 (18)	−0.0036 (13)	−0.0032 (14)	0.0016 (12)
O3	0.0188 (14)	0.0184 (13)	0.0209 (13)	0.0018 (11)	0.0031 (11)	−0.0011 (10)

### Geometric parameters (Å, °)

**Table:**

Fe1—O1W	2.137 (3)	O3W—H3B	0.821 (19)
Fe1—O2W	2.128 (3)	Fe2—C1	1.812 (4)
Fe1—O3W	2.098 (3)	Fe2—C2	1.807 (4)
Fe1—O1W^i^	2.137 (3)	Fe2—C3	1.808 (4)
Fe1—O2W^i^	2.128 (3)	Fe2—Br1	2.4479 (6)
Fe1—O3W^i^	2.098 (3)	Fe2—Br2	2.4605 (6)
O1W—H1A	0.821 (19)	Fe2—Br3	2.4558 (6)
O1W—H1B	0.821 (19)	C1—O1	1.126 (4)
O2W—H2A	0.821 (19)	C2—O2	1.128 (4)
O2W—H2B	0.821 (19)	C3—O3	1.129 (4)
O3W—H3A	0.821 (19)		
			
O3W—Fe1—O3W^i^	180.00 (18)	Fe1—O3W—H3A	113 (3)
O3W—Fe1—O2W^i^	89.97 (12)	Fe1—O3W—H3B	121 (3)
O3W^i^—Fe1—O2W^i^	90.03 (12)	H3A—O3W—H3B	109 (3)
O3W—Fe1—O2W	90.03 (12)	C2—Fe2—C3	91.43 (16)
O3W^i^—Fe1—O2W	89.97 (12)	C2—Fe2—C1	94.35 (15)
O2W^i^—Fe1—O2W	180.00 (16)	C3—Fe2—C1	95.59 (15)
O3W—Fe1—O1W	89.91 (10)	C2—Fe2—Br1	88.80 (11)
O3W^i^—Fe1—O1W	90.09 (10)	C3—Fe2—Br1	86.75 (11)
O2W^i^—Fe1—O1W	88.35 (10)	C1—Fe2—Br1	176.03 (11)
O2W—Fe1—O1W	91.65 (10)	C2—Fe2—Br3	87.49 (11)
O3W—Fe1—O1W^i^	90.09 (10)	C3—Fe2—Br3	177.51 (11)
O3W^i^—Fe1—O1W^i^	89.91 (10)	C1—Fe2—Br3	86.74 (11)
O2W^i^—Fe1—O1W^i^	91.65 (10)	Br1—Fe2—Br3	90.981 (19)
O2W—Fe1—O1W^i^	88.35 (10)	C2—Fe2—Br2	178.97 (12)
O1W—Fe1—O1W^i^	180.00 (14)	C3—Fe2—Br2	89.57 (11)
Fe1—O1W—H1A	107 (3)	C1—Fe2—Br2	85.77 (11)
Fe1—O1W—H1B	119 (4)	Br1—Fe2—Br2	91.04 (2)
H1A—O1W—H1B	109 (3)	Br3—Fe2—Br2	91.50 (2)
Fe1—O2W—H2A	128 (3)	O1—C1—Fe2	176.4 (3)
Fe1—O2W—H2B	121 (3)	O2—C2—Fe2	178.8 (3)
H2A—O2W—H2B	109 (3)	O3—C3—Fe2	178.4 (3)

Symmetry codes: (i) −*x*, −*y*, −*z*.

### Hydrogen-bond geometry (Å, °)

**Table:**

*D*—H···*A*	*D*—H	H···*A*	*D*···*A*	*D*—H···*A*
O1W—H1A···Br1	0.82 (2)	2.52 (2)	3.340 (3)	174 (4)
O1W—H1B···Br2^ii^	0.82 (2)	2.69 (2)	3.475 (3)	162 (5)
O2W—H2A···Br2^iii^	0.82 (2)	2.55 (2)	3.373 (3)	177 (5)
O2W—H2B···Br2^ii^	0.82 (2)	2.56 (2)	3.341 (3)	160 (5)
O3W—H3A···Br3	0.82 (2)	2.58 (2)	3.388 (3)	169 (5)
O3W—H3B···Br3^iv^	0.82 (2)	2.53 (2)	3.346 (3)	177 (4)

Symmetry codes: (ii) −*x*, −*y*+1, −*z*; (iii) *x*+1/2, −*y*+1/2, −*z*; (iv) −*x*+1/2, *y*−1/2, *z*.
